# Immunogenicity and Safety of a Live Attenuated Varicella Vaccine in Healthy Children Aged 12 to 15 Months: A Phase III, Randomized, Double-Blind, Active-Controlled Clinical Trial

**DOI:** 10.3390/vaccines13090973

**Published:** 2025-09-13

**Authors:** Nancy Nazaire-Bermal, Ningning Jia, Maria Angela C. Maronilla, Josemaria F. Lopez, Gang Zeng, Wenbin Wu, Adrielle Bernice C. Nimo, Chunfang Luan, Qianqian Xin

**Affiliations:** 1 San Juan De Dios Educational Foundation, Inc-Hospital, Pasay City 1300, Philippines; nnbermalmd@gmail.com (N.N.-B.); macmaronilla@gmail.com (M.A.C.M.); jololopez193@gmail.com (J.F.L.); abcn.abbi@gmail.com (A.B.C.N.); 2 Sinovac Holding Group Co., Ltd., Beijing 100085, China; jiann@sinovac.com (N.J.); zengg@sinovac.com (G.Z.); wuwb8122@sinovac.com (W.W.); 3 Sinovac (Dalian) Vaccine Technology Co., Ltd., Dalian 116620, China; luancf@sinovac.com

**Keywords:** varicella vaccine, immunogenicity, safety

## Abstract

**Objectives**: The varicella vaccine (VarV) produced by Sinovac (Dalian) obtained World Health Organization (WHO) prequalification in November 2022. However, no direct comparative studies have been conducted between VarV and other WHO-prequalified varicella vaccines. The study aimed to assess the immunogenicity and safety of Sinovac’s VarV compared with Merck Sharp & Dohme’s (MSD) VARIVAX^®^ (Moorgate, London, UK) following a single dose administration. **Methods**: This Phase III, randomized, double-blind, active-controlled, non-inferiority trial was conducted in the Philippines. Healthy children aged 12 to 15 months were enrolled. Eligible participants were randomly assigned (1:1) to receive a single dose of varicella vaccine either manufactured by Sinovac (Test group) or MSD (Active control group). Immunogenicity was evaluated 6 weeks after vaccination by enzyme-linked immunosorbent assay (ELISA). The primary immunogenicity endpoint was seroresponse rate 6 weeks after vaccination. Seroresponse rate was defined as varicella-zoster virus (VZV) antibody concentration ≥ 10 mIU/mL in participants who were seronegative (antibody concentration < 10 mIU/mL) at baseline. The secondary endpoint was the corresponding geometric mean concentration (GMC). Adverse events (AEs) and serious adverse events (SAEs) were monitored for 6 weeks after vaccination. **Results**: Among the 484 participants analyzed, the seroresponse rates 6 weeks after vaccination were 98.85% and 98.88% in the Test group and Active control group, respectively, with a difference of −0.03% (95% CI: −3.10%, 2.99%), which exceeded the predefined non-inferiority margin of −10%. The corresponding GMCs were 35.73 mIU/mL and 37.34 mIU/mL, respectively, with the ratio of 0.96 (95% CI: 0.86, 1.06), also exceeding the predefined non-inferiority margin of 0.67. Furthermore, the incidence of adverse reactions (ARs) in the Test group was lower than that in the Active control group (38.08% vs. 55.51%). **Conclusions**: Sinovac’s VarV demonstrated non-inferior immunogenicity to WHO-prequalified comparator vaccine (VARIVAX^®^) and favorable safety profile. These findings indicated that VarV (Sinovac, Beijing, China) met WHO standards for varicella vaccine evaluation, supporting its global use consideration.

## 1. Background

Varicella, caused by varicella-zoster virus (VZV), is a highly contagious disease primarily affecting children. Clinical manifestations include a characteristic vesicular rash, fever, and malaise [1,2]. Although often self-limiting, varicella can lead to severe complications, such as secondary bacterial infections, pneumonia, and encephalitis, particularly in immunocompromised individuals [3,4,5]. Following primary infection, VZV establishes latency and may reactivate as herpes zoster later in life [5].

Varicella vaccine (VarV), first developed in Japan in 1974, has proven highly effective in preventing varicella worldwide [6,7]. Clinical trials have demonstrated 70–90% efficacy in preventing infections and >95% protection against severe disease. As childhood immunization programs expand worldwide, particularly in developing countries, ensuring a stable supply of safe and effective varicella vaccines remains critical [8]. Among available options, VarV produced by Sinovac (Dalian) Vaccine Technology Co., Ltd., through cultivation in human diploid cells [9], obtained WHO prequalification in 2022, facilitating its global use.

In the pivotal Phase III clinical trial [10] involving 1–12 years old children, VarV (Sinovac) demonstrated 89.2% efficacy against varicella breakthrough cases and 100% efficacy against moderate or severe cases. Approximately 97% participants seroconverted after a single dose of VarV (Sinovac), with VZV antibody titers increased 7-fold compared with pre-vaccination. Additionally, large-scale applications confirmed the safety of VarV (Sinovac) [11,12]. All these findings supported VarV (Sinovac) as a valuable strategic option for varicella prevention and immunization programs supply.

In addition to VarV (Sinovac), several varicella vaccines have obtained WHO prequalification, including VARIVAX^®^ manufactured by MSD. Although most VarV are derived from the same strain, Oka strain, it has generally been assumed that all live attenuated varicella vaccines are likely to elicit comparable immune responses and provide similar protection against varicella [13,14]. Nevertheless, differences have been reported in the processes by which the vaccines are produced, and these differences may affect the degree of viral attenuation and the clinical performance of the vaccines [15]. According to the recommendations of World Health Organization (WHO), a head-to-head comparative study should be conducted to compare the immunogenicity and safety of VarV (Sinovac) with other WHO-prequalified VarVs.

Therefore, this Phase III, randomized, double-blind, active-controlled, non-inferiority trial was conducted to assess the immunogenicity and safety of a single dose of Sinovac’s VarV compared with Merck Sharp & Dohme’s (MSD) VARIVAX^®^ in healthy children

## 2. Methods

### 2.1. Study Design

Between 29 February and 6 September 2024, a Phase III randomized, double-blind, active-controlled, non-inferiority clinical trial was conducted in San Juan De Dios Educational Foundation Incorporated Hospital in the Philippines. Healthy children aged 12 to 15 months (*n* = 642) were enrolled and randomly assigned in a 1:1 ratio to receive a single dose of either the test VarV (Test group) or the active control vaccine, VARIVAX^®^ (Active control group). To facilitate participant compliance with study visits, participants were compensated for time and travel for each completed study visit. The study was approved by the Institutional Review Board (IRB) of the San Juan De Dios Educational Foundation Incorporated (IRB reference no: SJIRB-2023-0042/E-MED) and the Cardinal Santos Medical Center Ethics Review Committee (CSMC RERC, code: 2023-026). Good Clinical Practice (GCP) and the Declaration of Helsinki were followed during the trial. Written informed consent was obtained from all participants’ guardians before enrollment. The study was registered in Clinical Trials .gov on 23 January 2025 (NCT06314724). Following publication, the main results of this trial will be posted on the Clinical Trials. gov record.

### 2.2. Study Population

Healthy children between 12 and 15 months were enrolled in this study. The main inclusion criteria included: (1) a legal guardian able to understand and voluntarily sign informed consent; (2) able to comply with study procedures per investigator assessment; (3) provided verifiable identification and contact information.

Key exclusion criteria included: (1) prior vaccination with any varicella-containing vaccine or documented history of VZV infection; (2) known allergy to vaccines/vaccine ingredients or serious ARs to vaccines; (3) autoimmune diseases, immunodeficiency, immunosuppression, or asplenia; (4) receipt of any investigational vaccine within 30 days, live-attenuated vaccines within 28 days, or inactivated/subunit vaccines within 7 days prior to study vaccination; (5) acute illness or exacerbation of chronic disease within 7 days prior to vaccination; (6) axillary temperature > 37.0 °C at the time of vaccination; (7) any condition deemed by the investigator to interfere with results or increase participant risk.

Criteria for discontinuing a participant from further study procedures included the following: (1) Request from the participant’s guardian; (2) Occurrence of an intolerable adverse event (AE), regardless of whether it was related to the investigational product (IP); (3) The participant’s physical condition being deemed unsuitable for continued study participation; (4) Detection of any abnormal clinical symptoms by the investigator; (5) Other reasons as determined by the investigator. In cases where a participant withdraws from the study, data and blood samples collected before their withdrawal were still used in the analysis to maintain data integrity.

### 2.3. Vaccines

Detailed information of the test vaccine has been published previously. The comparator vaccine, VARIVAX^®^, contains a minimum of 1350 PFU (3.13 lg PFU) of Oka/Merck varicella virus per dose. All eligible participants received vaccines subcutaneously in the deltoid muscle region of the upper arm.

### 2.4. Immunogenicity Assessment

Blood samples (2.0–2.5 mL) were collected before vaccination and 6 weeks post-vaccination to evaluate immunogenicity [16].

VZV IgG antibodies were quantified using glycoprotein enzyme-linked immunosorbent assay (ELISA) kit. The enzyme-linked plates are pre-coated with purified VZV glycoproteins, which can specifically bind to the corresponding antibodies in serum samples. This method has undergone systematic methodological validation in strict accordance with the requirements of the Chinese Pharmacopoeia [1] and ICH Q2 (R2). The validation results confirm that this method meets the specified standards and can ensure the accuracy and stability of detection results.

All analyses were performed on the per-protocol set (PPS), including the randomized participants who met the inclusion criteria, did not meet the exclusion criteria, completed a single dose vaccination, and had valid immunogenicity data before and after vaccination. Seropositivity was defined as a VZV antibody concentration ≥10 mIU/mL. The susceptible and non-susceptible populations were defined as participants who were seronegative or seropositive at baseline, respectively.

The primary endpoint was seroresponse rate of VZV antibodies 6 weeks after vaccination in the susceptible population. Secondary endpoints included the GMC and geometric mean fold rise (GMFR) 6 weeks after vaccination in the same population.

### 2.5. Safety Assessment

Parents/guardians were provided with diary cards and instructed on recording AEs after vaccination. All safety analyses were performed on the safety set (SS), which included all randomized participants, given a single dose of vaccine, and had at least one safety evaluation information. After vaccination, all participants must be monitored on site for 30 min. Solicited local AEs (e.g., pain, induration, swelling, erythema, rash, and pruritus) and solicited systemic AEs (e.g., pyrexia, acute allergic reaction, diarrhea, nausea, vomiting, and cough) were actively monitored for 14 days following vaccination. Study investigators conducted a face-to-face interview on day 15 to ensure completeness and accuracy of the safety data. Unsolicited AEs and serious adverse events (SAEs) were followed up for 42 days after vaccination. Trial monitoring was performed weekly by monitors to verify data against source documents and ensure adherence to the protocol. The causal relationship between AEs and vaccination were judged by investigators.

The grading standard for ARs was based on the “Guidelines for Adverse Event Classification Standards for Clinical Trials of Preventive Vaccines” of NMPA in China [17].

### 2.6. Randomization and Masking

After enrollment, eligible participants were randomly assigned in a 1:1 ratio to receive either the test vaccine (VarV, Sinovac) or the active control vaccine (VARIVAX^®^, MSD). Randomization was conducted using Statistical Analysis System (SAS) 9.4 software to create unique random codes, with all vaccines subsequently relabeled by unmasked personnel not involved in clinical assessments according to these codes to uphold double-blind conditions. Enrolled participants were randomly assigned matching codes corresponding to their vaccine group allocation. Throughout the study, participants, investigators, outcome assessors, and sponsor representatives remained blinded to treatment assignment.

Vaccine administration and unblinding procedures adhered strictly to the pre-established randomization scheme. In the event of a medical emergency requiring knowledge of the vaccine identity, emergency unblinding was performed via the Interactive Web Response System (IWRS) after agreement between the principal investigator and the sponsor. The reason for unblinding was documented, and the unblinded participant was discontinued from the trial. Full study unblinding occurred after database lock and completion of all laboratory assays and safety follow-up.

### 2.7. Sample Size Determination

Sample size determination was based on VZV antibody responses following single-dose vaccination, considering both seroresponse rate and GMC at 6 weeks post-vaccination. Using the Miettinen and Nurminen method for the primary hypothesis of seroresponse rate, calculations incorporated a one-sided α of 0.025, β of 0.1, non-inferiority margin of −10%, expected seroresponse rate of 90%, and 1:1 sample ratio, yielding 205 participants per group. For the secondary hypothesis of VZV antibody GMC, calculations used similar parameters with a GMC standard deviation of 0.5 and a non-inferiority margin of 0.67, requiring 171 participants per group. According to the principle of maximizing sample size, the sample size calculation based on seroresponse rate is the largest. Meanwhile, considering approximately 20% loss of follow-up and 80% susceptible participants, 321 participants will be needed for both the Test group and the Active control group.

### 2.8. Statistical Analysis

The statistical criterion was based on non-inferiority, which required that the lower limit of the 95% confidence interval (CI) of the difference (VarV-VARIVAX^®^) of seroresponse rate ≥ −10%; meanwhile, the lower limit of the two-sided 95% CI for the ratio (VarV/VARIVAX^®^) of GMC was >0.67. Two-sided 95% CIs were calculated using the Clopper-Pearson method. Inter-group comparisons utilized the Chi-square and Fisher’s exact tests. For the GMC and GMFR analyses, the LS-adjusted log-transformed values were derived using the analysis of covariance model to account for potential baseline imbalances and enhance the robustness of the comparisons. All hypothesis testing employed two-sided significance testing, considering *p* values less than 0.05 as statistically significant.

In immunogenicity analysis, the missing values in post-vaccination antibody data were imputed by using LOCF (Last Observation Carried Forward) method. The missing data in safety evaluation were not be imputed. All AEs were coded using the Medical Dictionary for Regulatory Activities (MedDRA), version 26.0.

## 3. Results

### 3.1. Participants Included in the Study

**Figure 1:**
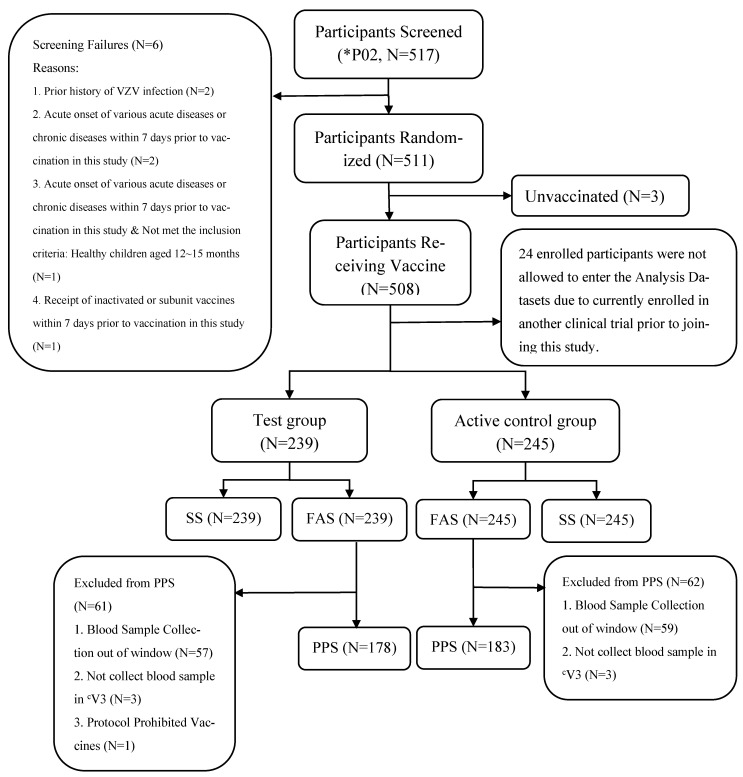
Disposition of Participants. * P01 site’s data were excluded from the analysis due to protocol violations and concerns about the authenticity of birth documentation. ^a^ V1: On the day of vaccination. ^b^ V2: V1 + 14 days (+7 days), Safety assessment. ^c^ V3: V1 + 42 days (+14 days), Immunogenicity assessment, V1 + 42 days (+14 days).

A total of 642 participants were initially enrolled across two centers in the Philippines: Fe del Mundo Medical Center (P01, *n* = 131) and San Juan de Dios Educational Foundation Incorporated Hospital (P02, *n* = 511). According to the Philippines Food and Drug Administration’s decision, all data from the P01 site were excluded from the analysis due to protocol violations, including concurrent trial enrollment (*n* = 32) and concerns about the authenticity of birth documentation (*n* = 40). In the P02 site, 3 participants did not receive vaccination, and 24 were excluded due to concurrent trial enrollment. Although these exclusions resulted in a sample size smaller than the originally planned requirement, baseline characteristics remained balanced between groups, and the non-inferiority conclusion was not affected. Finally, the analysis included 484 participants for analysis (Figure 1), including 239 in the Test group and 245 in the Active control group.

The demographic characteristics concerning age, gender, race distribution, height, and weight were similar between the Test group and the Active control group. Details are shown in Table 1 Demographic and other baseline characteristics in FAS/SS.

### 3.2. Immunogenicity Results

Before vaccination, seropositivity rates were 2.25% for the Test group and 2.73% for the Active control group, with GMCs of 2.68 mIU/mL and 2.78 mIU/mL, respectively. Both seropositivity rates and GMCs were comparable between the two groups. For the primary endpoint, the seroresponse rates at 42 days post-vaccination among the susceptible population were 98.85% for the Test group and 98.88% for the Active control group, with a difference of −0.03% (95% CI: −3.10%, 2.99%). The seroresponse rate in the Test group met the non-inferiority criteria, confirming the achievement of the primary endpoint.

**Figure 2:**
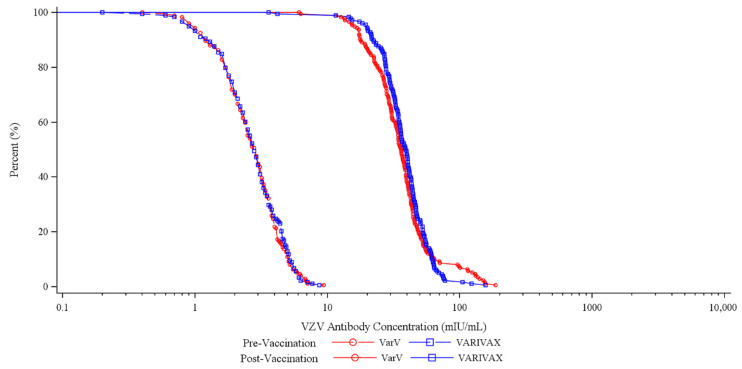
Inverse distribution curve for VZV antibody titers in the susceptible population.

Regarding the secondary endpoint, the corresponding GMCs for the Test group and Active control group were 35.73 mIU/mL and 37.34 mIU/mL, respectively, yielding a ratio of 0.96 (95% CI: 0.86, 1.06). The GMC in the Test group met the non-inferiority criteria, indicating that the secondary endpoint was achieved. Additionally, the GMCs significantly increased 42 days post-vaccination compared to pre-vaccination, with GMFRs of 13.96 in the Test group and 14.55 in the Active control group. These details are presented in Table 2. The inverse distribution curve for VZV antibody titers in susceptible populations is illustrated in Figure 2.

After a single dose of, the seroresponse rate and GMC in the Test group were non-inferior to those in the Active control group, and both primary and secondary endpoints were achieved. These findings demonstrate that VarV (Sinovac) exhibits excellent immunogenicity and is non-inferior to VARIVAX^®^.

### 3.3. Safety Results

The overall incidence of AEs within 42 days after vaccination was comparable in the Test group and the Active control group, at 63.60% and 72.24%, respectively. However, the incidence of AR in the Test group was significantly lower than that in the Active control group (*p* = 0.0001), with most ARs occurring within 14 days after vaccination at rates of 38.08% and 55.51%, respectively. The majority of reported ARs were grade 1 and grade 2. The incidences of grade 1, 2 and 3 ARs were 32.64%, 10.04%, and 1.67% for the Test group, and 51.43%, 13.88%, and 2.04% for the Active control group, respectively. The incidences of grade 2 and grade 3 ARs were comparable between the two groups, while the incidence of grade 1 ARs in the Test group was significantly lower than that in the Active control group (*p* < 0.0001). Details are shown in Table 3.

The most frequent ARs were general disorders and administration site conditions, with the incidence in the Test Active control group being lower than in the Active control group (31.38% vs. 51.84%). The very common ARs (≥10%) in the Test group included pyrexia and erythema at the vaccination site. The incidences of pyrexia were comparable between the two groups (15.48% vs. 18.37%). In contrast, the incidence of vaccination site erythema in the Test group was significantly lower than in the Active control group (15.06% vs. 40.41%).

A total of 5 participants (2.09%) in the Test group reported 7 SAEs, while 8 participants (3.27%) in the Active control group reported 9 SAEs, which were comparable between the two groups. Importantly, no SAEs related to vaccination were reported in the Test group, while 0.82% (2/245) were reported in the Active control group.

## 4. Discussion

This randomized, double-blind, active-controlled trial demonstrates that VarV manufactured by Sinovac (Dalian) exhibits non-inferior immunogenicity and superior safety compared to the WHO-prequalified VARIVAX^®^ in healthy children aged 12–15 months.

In the study, both the primary and secondary endpoints were achieved. For the primary endpoint, the seroresponse rates were high, exceeding 98% in both the Test group and the Active control group. The corresponding difference was −0.03% (95% CI: −3.10%, 2.99%), indicating that the immunogenicity of VarV (Sinovac) was non-inferior to that of VARIVAX^®^. Regarding the secondary endpoint, the GMCs were comparable between the two groups (35.73 mIU/mL vs. 37.34 mIU/mL), with the ratio (VarV (Sinovac) /VARIVAX^®^) of 0.96 (95% CI: 0.86, 1.06). The analysis further supported the non-inferiority of VarV (Sinovac). These findings demonstrated that the immunogenicity of VarV manufactured by Sinovac was comparable to that of the WHO pre-qualified VarV, VARIVAX^®^, in this pediatric population. In comparison, the fold increase of the antibody levels observed in the Test group among susceptible populations in this study was relatively higher than those reported in previous studies of VarV (Sinovac) (approximately 5 to 9-fold rise) [18,19,20]. This discrepancy might be attributed to variations in the assay methods used for antibody detection.

Additionally, the results suggested that the safety profile of VarV (Sinovac) might be superior to that of the active-control vaccine. Comparatively, the overall incidence of ARs in the Test group was statistically significantly lower than in the Active control group, with incidences of 38.08% and 55.51%, respectively. This disparity is primarily attributed to the difference in the incidence of vaccination site erythema (15.06% vs. 40.41%). In previous studies of VarV (Sinovac), the incidences of vaccination site erythema were less than 1% [19,21], while the incidence of VARIVAX^®^ exceeded 30% in published clinical data [22,23]. The literature results were essentially in line with the current study’s findings, despite variations in the methods of safety data collection among different clinical trials. More importantly, there were statistically significant differences in the severity of vaccination site erythema between the two groups, particularly for grade 2. The incidence of grade 2 vaccination site erythema in the Active control group was 10-fold higher than that in the Test group, with incidences of 4.08% and 0.42%, respectively. Most ARs in the Test group occurred close to the vaccination and were mild or moderate, which was consistent with previous studies [2]. The incidences of SAEs were comparable between the two groups, with 2.09% in the Test group and 3.27% in the Active control group. No SAEs related to vaccination were reported in the Test group, whereas 0.82% were reported in the Active control group, further underscoring the favorable safety profile. Overall, VarV (Sinovac) demonstrates non-inferior immunogenicity to VARIVAX^®^ with a significantly improved safety profile.

The current study confirms the robust short-term safety and immunogenicity profile of the VarV (Sinovac) vaccine following a single dose. Nonetheless, these findings should be interpreted within the broader context of long-term protection and evolving global vaccination strategies. The single-dose strategy demonstrated moderate effectiveness (~83%) [24] against any varicella, though it is often associated with breakthrough infections due to suboptimal immunogenicity in some individuals and waning immunity over time. In contrast, a two-dose regimen, which has been adopted by a growing number of countries, show higher effectiveness (~95%) [25] and markedly reduces breakthrough infections by inducing higher and more durable antibody titers [26].

As emphasized by the World Health Organization (WHO), the number of doses recommended is dependent on the specific goals of the vaccination programme [27]. A single dose is sufficient to reduce mortality and severe morbidity from varicella. In contrast, a two-dose regimen is recommended for countries whose programmatic goal extends to further reduce the number of cases and outbreaks. In those with a high burden of varicella disease, the public health priority remains achieving high coverage (>80%) to reduce disease incidence and transmission. Our results, which demonstrate strong immunogenicity and well-stablished safety after a single dose, provide a solid foundation for protection against varicella and support the feasibility of this initial strategic goal.

Several limitations should be considered in this study. Firstly, protocol deviations led to the exclusion of some participants data, resulting in the final sample size smaller than originally planned. However, the comparability of baseline characteristics and VZV antibody levels between the study groups, as well as the demonstration of non-inferiority, indicated that the data exclusion did not have a substantial impact on the primary endpoint assessment. Secondly, the follow-up duration was limited to 42 days after primary immunization, which restricts insight into long-term immune persistence. Finally, this trial was conducted under controlled conditions. Real-world data are still needed to confirm the effectiveness of VarV (Sinovac) in routine practice. Future research will prioritize the evaluation of the immune persistence and effectiveness.

## 5. Conclusions

This clinical trial provides robust evidence that the immunogenicity of VarV (Sinovac), manufactured by Sinovac, was comparable to that of the WHO pre-qualified VarV, VARIVAX^®^, in healthy children aged 12 to 15 months. Moreover, VarV (Sinovac) demonstrated favorable safety profile. These findings support its suitability for global immunization programs.

## Figures and Tables

**Table 1:** Demographic and other baseline characteristics in FAS/SS.

Indicator	Test Group	Active Control Group	*p* Value
No.	239	245	
Age (Months), Mean ± SD	13.2 ± 1.064	13.1 ± 1.015	0.2044
Gender			0.7048
Male, *n* (%)	128 (53.56)	127 (51.84)	
Female, *n* (%)	111 (46.44)	118 (48.16)	
Race			1.0000
Asian, *n* (%)	239 (100.00)	244 (99.59)	
White, *n* (%)	0 (0.00)	1 (0.41)	
Height(cm), Mean ± SD	73.71 ± 2.973	73.95 ± 3.399	0.4087
Weight(kg), Mean ± SD	8.82 ± 1.240	8.92 ± 1.288	0.3956

**Table 2:** Immunogenicity results of participants on day 42 after vaccination in the susceptible population.

Indicator	Test Group (N = 175)	Active Control Group (N = 178)	* Difference/Ratio (95%CI)	*p* Value
Pre-vaccination				
GMC	2.56	2.57		0.9509
(95% CI)	(2.35, 2.78)	(2.35, 2.80)		
Post-vaccination				
Seroresponse rate, (%)	98.85	98.88	−0.03	1.0000
(95% CI)	(95.91, 99.86)	(96.00, 99.86)	(−3.10, 2.99)	
GMC	35.73	37.34	0.96	0.4103
(95% CI)	(33.15, 38.51)	(34.68, 40.21)	(0.86, 1.06)	
GMFR	13.96	14.55		0.5786
(95% CI)	(12.56, 15.52)	(13.16, 16.07)		

* The difference of seroresponse rates and the ratio of GMCs were calculated between the Test group and the Active Control group, respectively.

**Table 3:** Vaccine-related AEs occurred within 42 days after vaccination.

	Test Group (N = 239)		Active Control Group (N = 245)		*p* Value
ARs	No. of Events	No. of Participants (%)	No. of Events	No. of Participants (%)	
**Overall**	170	91 (38.08)	253	136 (55.51)	0.0001
Grade 3	4	4 (1.67)	11	5 (2.04)	1.0000
**Solicited**	157	89 (37.24)	238	136 (55.51)	<0.0001
**Local**					
Pain	17	17 (7.11)	26	26 (10.61)	0.2024
Induration	2	2 (0.84)	1	1 (0.41)	0.6196
Swelling	2	2 (0.84)	7	7 (2.86)	0.1759
Erythema	36	36 (15.06)	99	99 (40.41)	<0.0001
Rash	0	0 (0.00)	1	1 (0.41)	1.0000
Pruritus	2	2 (0.84)	1	1 (0.41)	0.6196
**Systemic**					
Pyrexia	40	37 (15.48)	51	45 (18.37)	0.4673
Hypersensitivity	1	1 (0.42)	0	0 (0.00)	0.4938
Diarrhea	21	20 (8.37)	18	17 (6.94)	0.6098
Nausea	5	5 (2.09)	4	4 (1.63)	0.7489
Vomiting	10	10 (4.18)	10	9 (3.67)	0.8184
Cough	21	19 (7.95)	21	18 (7.35)	0.8650
**Unsolicited**	13	11 (4.60)	15	13 (5.31)	0.8350

## Data Availability

Individual participant data are available under restricted access for the requirements imposed by the Chinese Human Genetic Resources Administration concerning the public disclosure of clinical trial data. Researchers who provide a scientifically sound proposal will be allowed to access the de-identified individual participant data. Individual participant data can be obtained with a re-quest to the corresponding author (xinqq@sinovac.com).
